# The increase in the surface brightness of the night sky and its importance in visual astronomical observations

**DOI:** 10.1038/s41598-023-44423-w

**Published:** 2023-10-10

**Authors:** Marcin Wesołowski

**Affiliations:** College of Natural Sciences, Institute of Physics, University of Rzeszów, Pigonia 1 Street, 35-310 Rzeszów, Poland

**Keywords:** Astronomy and astrophysics, Planetary science

## Abstract

The problem of sky pollution with artificial light currently affects practically all branches that are related to the broadly understood environment. This is especially true for astronomical observations. This paper presents the results of measurements of the surface brightness of the night sky for the city of Rzeszów and the surrounding area, which were made in 2015, 2018 and 2021 using the photometer Sky Quality Meter (SQM-L). The measurements show that the surface brightness of the night sky in Rzeszów in 2015 was in the range from 19.20 to 18.67 mag/arcsec^2^, in 2018 the value of brightness oscillated in the range from 18.53 to 16.47 mag/arcsec^2^, and in 2021 this value was in the range from 17.13 by 15.11 mag/arcsec^2^. Translating the obtained values on the Bortle scale, we can see an increase in the brightness of the night sky from class VI in 2015 to class IX in 2021. A similar trend applies to neighboring towns, for which an increase in brightness from class IV in 2015 to class VIII in 2021 was also observed. An increase in the surface brightness of the night sky causes a deterioration or even loss of visibility of astronomical objects in the night sky. This is especially true for comets and low-brightness stars. Based on the measurement results, a forecast of the development of light pollution was prepared for the study areas until 2025. This forecast shows that we will still have to deal with an increase in the surface brightness of the night sky. Due to the further projected increase in the brightness of the night sky, it should be expected that observers in small towns will experience changes in the quality of the sky over the coming years, which will undoubtedly make astronomical observations difficult, in particular for faint celestial bodies such as comets.

## Introduction

The problem of sky pollution by artificial light plays a fundamental role in astronomical observations in particular. The direct cause of this phenomenon can be distinguished by two basic factors, natural or artificial. In the case of natural factors, the surface brightness of the sky (hereinafter referred to as sky brightness) consists of the brightness coming from celestial bodies: the Moon, planets, comets and stars. In turn, artificial factors are related to broadly understood human activity, including the urbanization of increasingly larger areas. Light pollution of the night sky with artificial light is a problem that occurs in virtually all countries around the world. This applies in particular to highly developed and rapidly developing countries. In Europe, an example of such a country is Poland, where there is practically no longer a naturally dark place free from light pollution. The exception to this rule is two dark sky parks (Bieszczady Dark Sky Park and Izerski Dark Sky Park) and regional associations established to protect naturally dark sky. Therefore, it is extremely important to conduct systematic, local monitoring of the surface brightness of the night sky in the context of visual astronomical observations. In addition, this phenomenon reduces the contrast between the observed celestial body and the naturally dark night sky. This results in the limitation of the visibility of celestial bodies, especially near large cities, as well as the dynamic development of the light island. Consequently, even at considerable distances from the city center, astronomical observations of faint bodies are practically impossible due to residual light pollution. Note that in areas with dark skies, the human eye can see a star with an apparent brightness of up to + 6 magnitude. In the case of naturally dark areas, the limit of the visibility of astronomical objects for an average person would be a star with an apparent brightness of + 8 magnitude^1^. To assign the brightness of the sky to a given class, the Bortle 9-point scale^2,3^ is commonly used, which is presented in Table 1.

**Table 1:** Bortle's classification in relation to the surface brightness of the night sky.

Class	Type of sky	Values SQM mag/arcsec^2^)	NELM (mag)	The color of the sky
I	Excellent dark-sky site	21.75–22.0	7.6–8.0	Black
II	Typical a truly dark site	21.60–21.75	7.1–7.5	Gray
III	Rural sky	21.30–21.60	6.6–7.0	Blue
IV	Brighter rural/suburban transition sky	20.30–21.30	6.1–6.5	Green/yellow
V	Suburban sky	19.25–20.30	5.6–6.0	Orange
VI	Bright suburban sky	18.50–19.25	5.1–5.5	Red
VII	Suburban/urban transition	18.00–18.50	4.6–5.0	Red
VIII	City sky	< 18.00	4.1–4.5	White
IX	Inner-city sky	< 18.00	< 4.0	White

Based on this classification, we can determine the naked-eye visibility of astronomical objects in the night sky. In addition, for each class, the range of visibility for the NELM expressed in magnitude was given.

In addition, light pollution negatively affects human health and safety, ecology and economy, culture and aesthetics^4,5^. All this makes the phenomenon of light pollution a very serious problem giving negative knocks similar to the pollution of water, air, soil and atmosphere. However, this phenomenon in relation to these commonly known types of pollution is a problem that receives only a little attention. Note that the effects of light pollution have been the subject of research for over 30 years, especially among astronomers^6–22^.

The research results presented in this paper are a continuation of my measurements related to local pollution of the night sky, which is caused by artificial light. The first paper presents the results of measurements of the surface brightness of the night sky in 2015 and 2018^5^. The results of measurements from 2021 and the forecast of the development of the phenomenon until 2025 have been added to the current paper. The obtained measurement results were used to determine the condition of the visibility of comets with the naked eye in the night sky.

## Method

When measuring the brightness of the sky, the Sky Quality Meter^13,23^ was used, which is available in two versions: SQM or SQM-L. It is a simple photometer that measures radiance^23^, i.e. the amount of light emerging from a certain sky area and reaching the sensor surface. The radiance is automatically converted to a unit of surface brightness (mag/arcsec^2^). In the case of the SQM-L version, we additionally have a built-in lens that provides a measurement range limited to 20° (in the SQM version, this range is equal to 84°). Then we can make more precise measurements of the selected part of the sky because the sensor does not collect light from the horizon, where there is usually more light pollution. The accuracy of measuring the brightness of the night sky is equal to ± 10% of the measured value^24^. In addition, in the context of the accuracy of the measurements taken, it is extremely important to take into account the effects of sensor ageing, i.e. deterioration of its sensitivity or even loss of optical elements transmission^25^.

**Figure 1:**
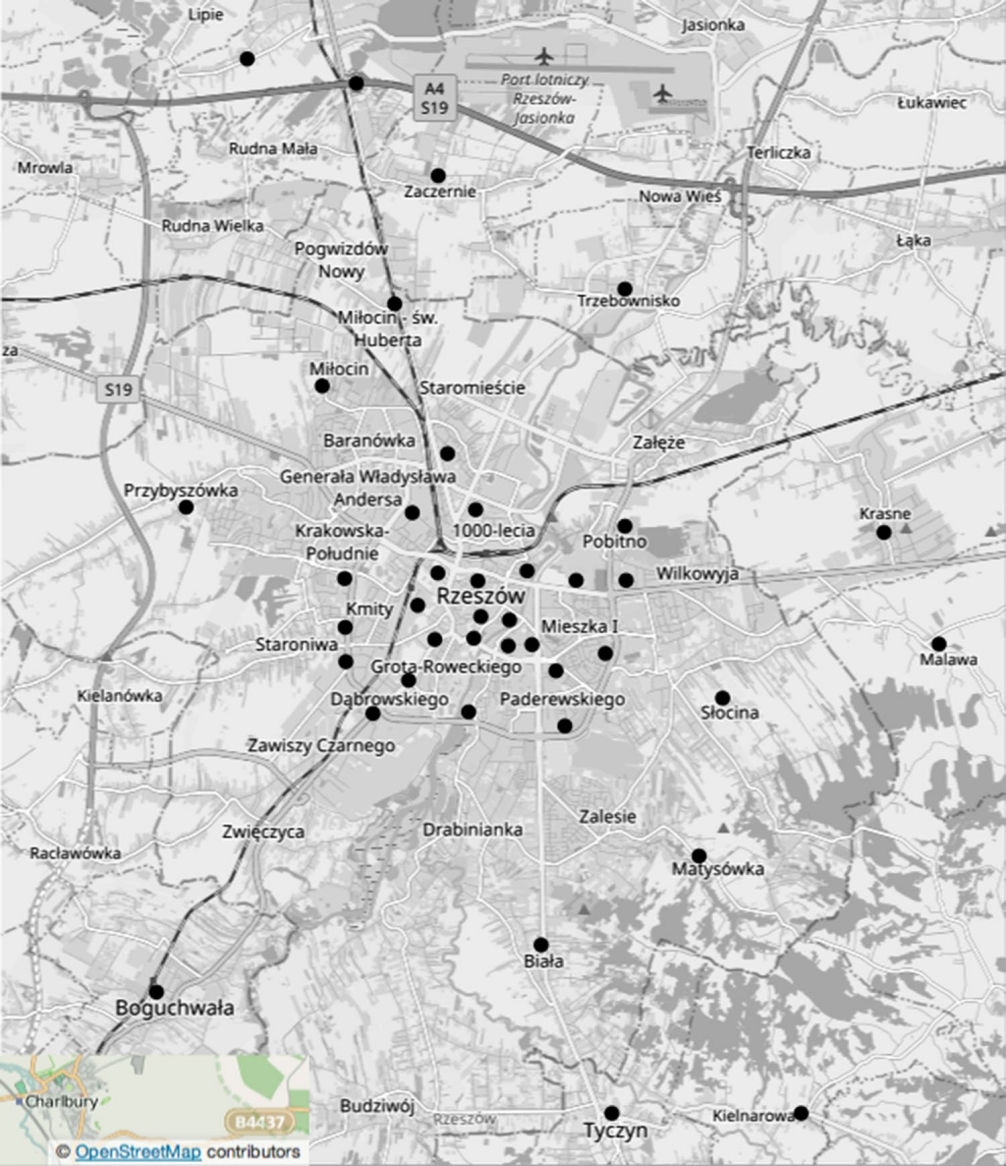
Distribution of points where the measurements were carried out in the city of Rzeszów and the neighboring towns. The map was generated based on the website: http://www.OpenStreetMap.org and the measurement points were marked in the Inkscape graphics program, which is a free, open-source vector graphics editor based on the General Public License.

The measurement result is therefore the sum of the total amount of light reaching the photometer window through its field of view. In order to eliminate the influence of the observer's micro-movements (e.g. hand vibrations) during the measurements, the device was placed on a photographic tripod. For the measurements to correspond to the real values for the city center, 25 measurement points were selected. In the case of neighboring towns, the number of measurement points was 15. Measuring points where the research was carried out were located in individual districts of the city and neighboring towns. The arrangement of these points is shown in Fig. 1. Let us add that at each of these points, the measurement procedure was the same way and the measurements were recorded in a paper form to a previously prepared table.

During the measurements, the SQM-L meter was set in the direction of the zenith. Measurements were taken during the new moon to eliminate its influence on the total surface brightness of the night sky. In the years covered by the study, the measurements were regular (at least 8 times a year) in cloudless sky. On average, several measurements were recorded at each measurement point, and the measurement results presented in the paper are averaged values. When analyzing the measurement results, it should be noted that with a dark sky, the SQM-L photometer registers a higher numerical value of the surface brightness of the night sky. Similarly, when the sky is brighter, e.g. in the city center, the photometer returns a numerically lower measurement value. It should be noted that the brightness scale used is consistent with the brightness scale of astronomical objects.

## Results

**Figure 2:**
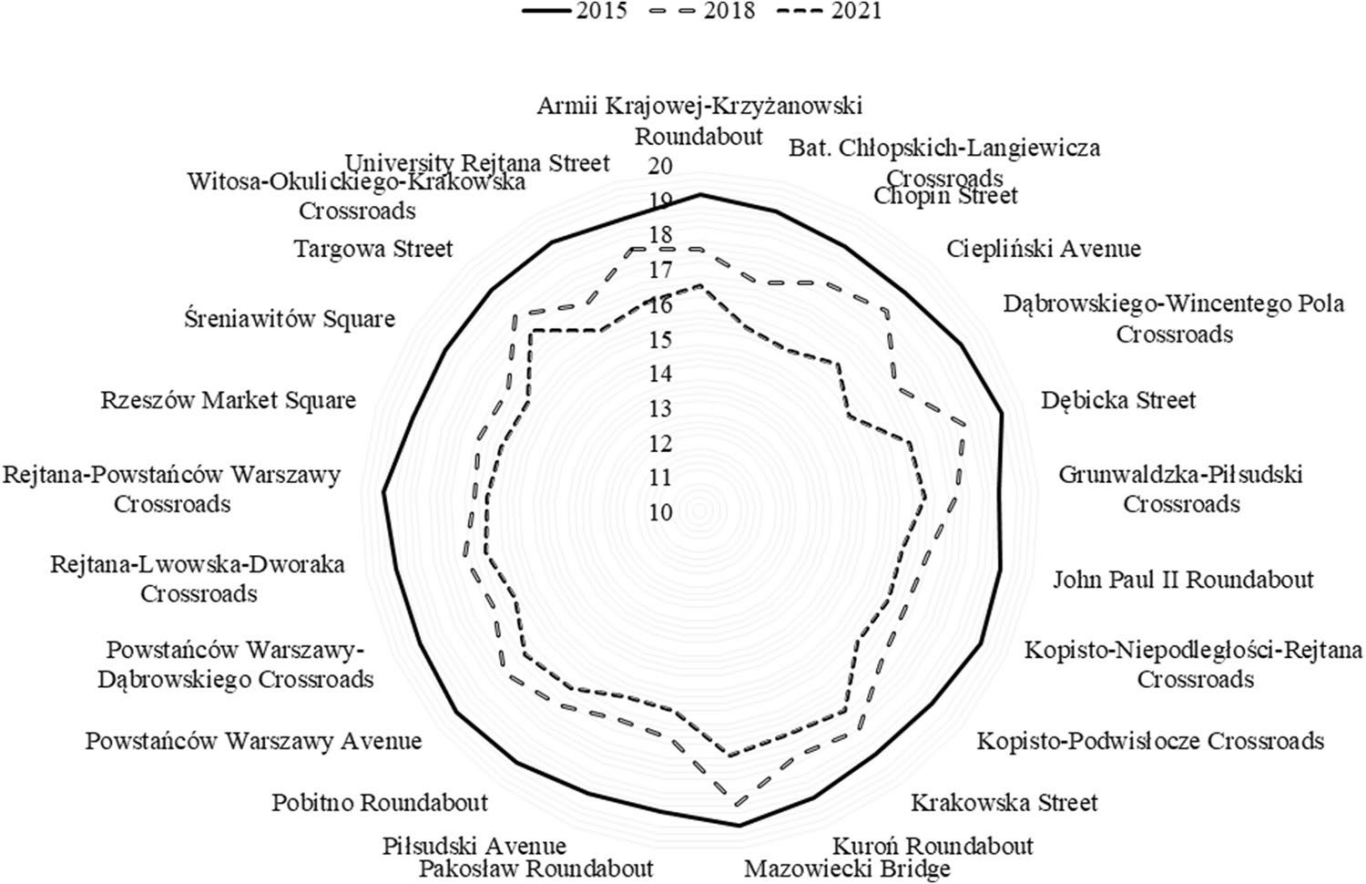
Distribution of surface brightness of the night sky in Rzeszów in 2015–2021. The numbering of points on the graph is consistent with Table 2.

**Figure 3:**
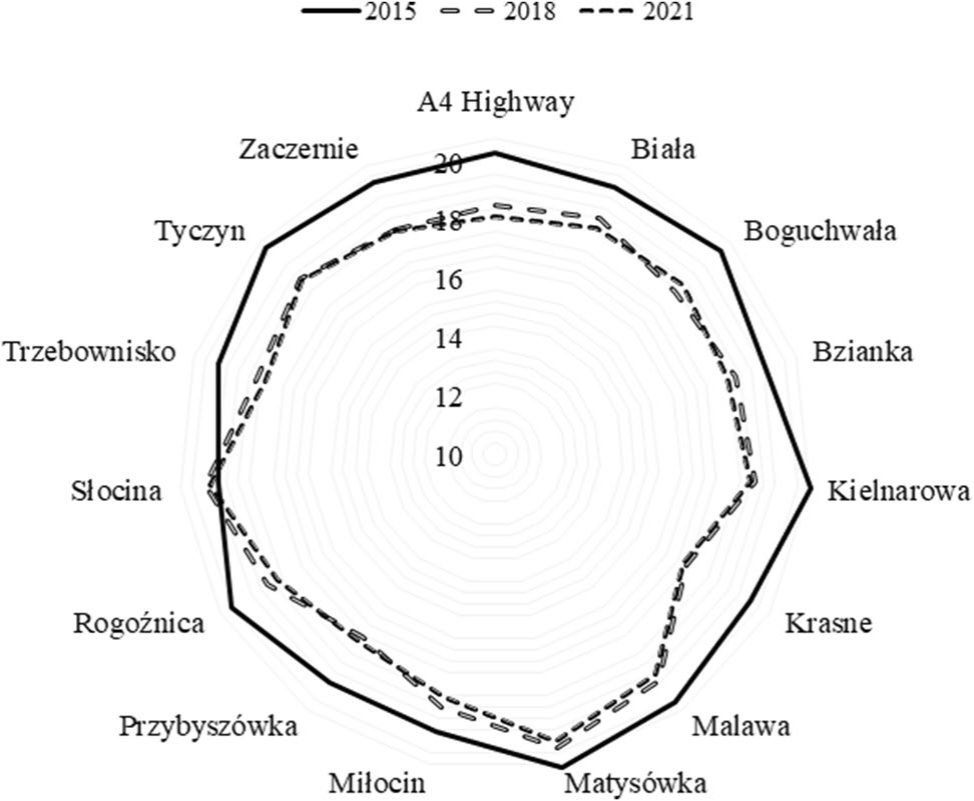
Distribution of surface brightness of the night sky in neighboring towns in 2015–2021. The numbering of points on the graph is consistent with Table 3.

**Figure 4:**
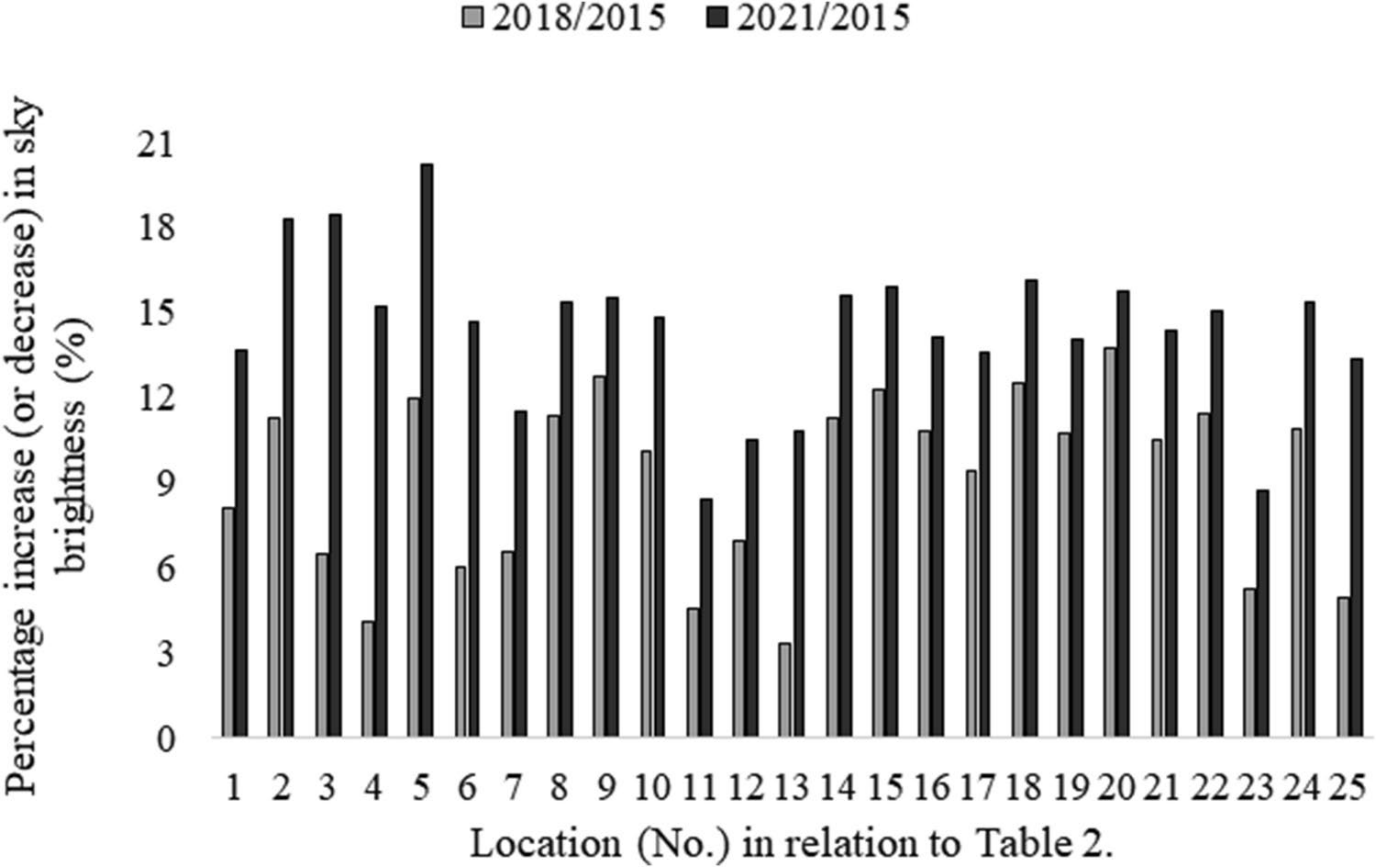
Percentage change in the surface brightness of the night sky in Rzeszów in two consecutive measurement series (2018, 2021) in relation to 2015.

**Figure 5:**
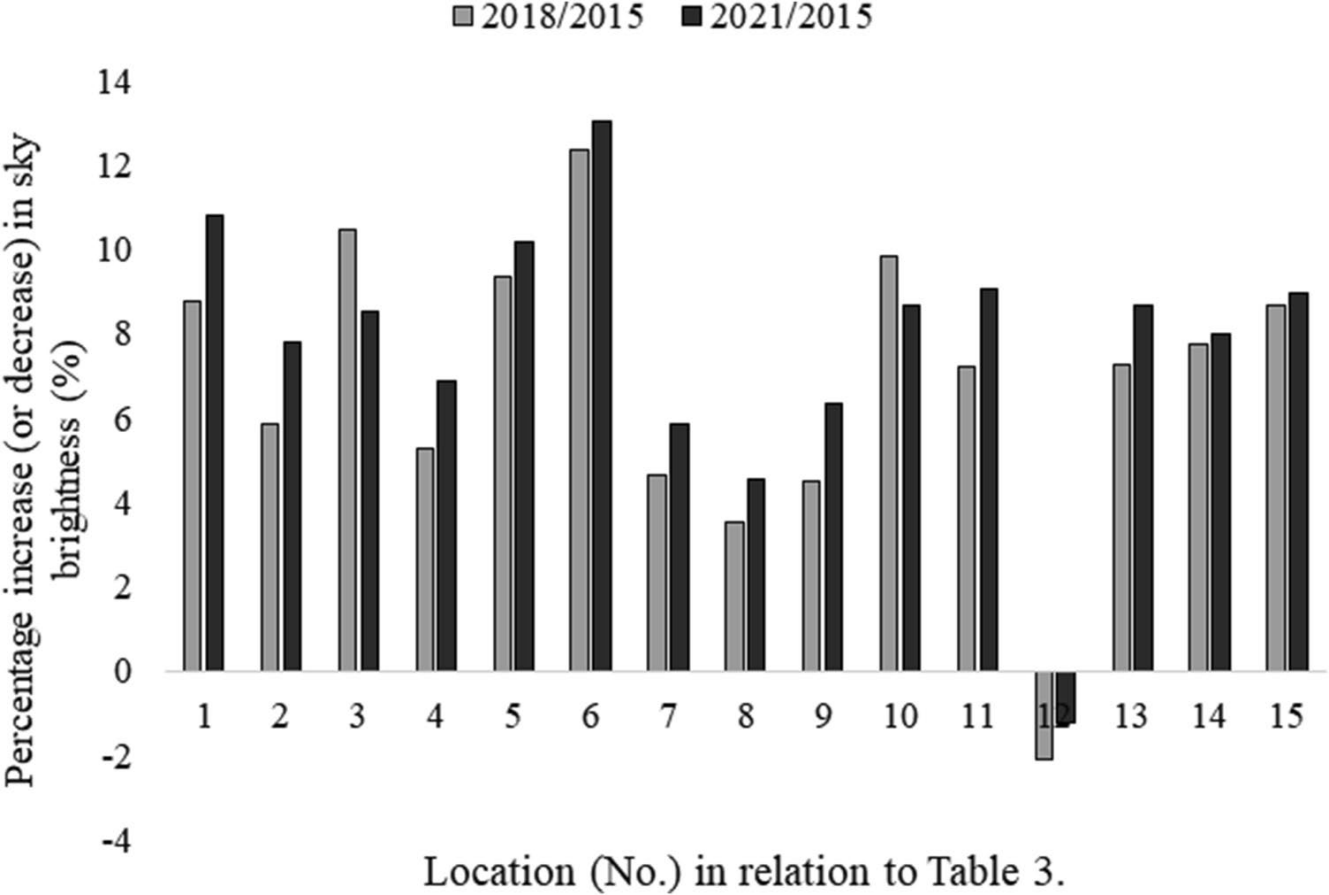
Percentage change in the surface brightness of the night sky in the neighboring towns in two consecutive measurement series (2018, 2021) compared to 2015. Note that for measurement point 12, based on Table 3, a negative value means a decrease in the surface brightness of the night sky.

The paper presents the results of measurements of the surface brightness of the sky together with the forecast of the development of the phenomenon for the city of Rzeszów and the surrounding area. For this purpose, 40 points were identified (25 in the city and 15 in neighboring towns) where measurements were carried out. The obtained measurement results show, that despite the increasing awareness of the local society, the brightness of the sky is unfortunately still worsened. The measurement results were grouped for the city center and the results are presented in Table 2. In the case of neighboring towns, the measurement results are presented in Table 3. The graphic distribution of measurements of changes in the brightness of the night sky of Rzeszów and the surrounding area in the time frames covered by the study is presented in Figs. 2, 3. The first measurements of the surface brightness of the night sky in Rzeszów and neighboring towns were carried out in 2015^5^. To determine the behavior and the trend of the phenomenon, two percentage ratios were determined based on subsequent measurement series 2018/2015 and 2021/2015. The results of this analysis are shown in Figs. 4, 5.

**Table 2:** The results of measurements of the night sky's brightness in Rzeszów in 2015, 2018 and 2021.

No.	Location	Brightness (mag/arcsec^2^)		
		2015	2018	2021
1	Armii Krajowej-Krzyżanowski (R)	19.13 ± 1.91	17.58 ± 1.76	16.52 ± 1.65
2	Bat. Chłopskich-Langiewicza (C)	18.93 ± 1.89	16.79 ± 1.68	15.47 ± 1.55
3	Chopin (S)	18.74 ± 1.87	17.53 ± 1.75	15.29 ± 1.53
4	Ciepliński (A)	18.68 ± 1.87	17.92 ± 1.79	15.84 ± 1.58
5	Dąbrowskiego-Wincentego Pola (C)	18.94 ± 1.89	16.68 ± 1.67	15.11 ± 1.51
6	Dębicka (S)	19.20 ± 1.92	18.05 ± 1.81	16.38 ± 1.64
7	Grunwaldzka-Piłsudski (C)	18.67 ± 1.87	17.44 ± 1.74	16.52 ± 1.65
8	John Paul II (R)	18.83 ± 1.88	16.70 ± 1.67	15.94 ± 1.59
9	Kopisto-Niepodległości-Rejtana (C)	18.97 ± 1.89	16.56 ± 1.66	16.02 ± 1.60
10	Kopisto-Podwisłocze (C)	18.70 ± 1.87	16.81 ± 1.68	15.93 ± 1.59
11	Krakowska (S)	18.68 ± 1.87	17.83 ± 1.78	17.11 ± 1.71
12	Kuroń (R)	18.92 ± 1.89	17.61 ± 1.76	16.93 ± 1.69
13	Mazowiecki Bridge	19.17 ± 1.92	18.53 ± 1.85	17.10 ± 1.71
14	Pakosław (R)	18.74 ± 1.87	16.63 ± 1.66	15.82 ± 1.58
15	Piłsudski (A)	18.77 ± 1.87	16.47 ± 1.65	15.78 ± 1.58
16	Pobitno (R)	18.98 ± 1.89	16.93 ± 1.69	16.30 ± 1.63
17	Powstańców Warszawy (A)	19.12 ± 1.91	17.32 ± 1.73	16.52 ± 1.65
18	Powstańców Warszawy-Dąbrowskiego (C)	18.94 ± 1.89	16.57 ± 1.66	15.88 ± 1.59
19	Rejtana-Lwowska-Dworaka (C)	18.93 ± 1.89	16.90 ± 1.69	16.27 ± 1.63
20	Rejtana-Powstańców Warszawy (C)	19.15 ± 1.92	16.52 ± 1.65	16.14 ± 1.61
21	Rzeszów Market (Sq)	18.70 ± 1.87	16.73 ± 1.67	16.02 ± 1.60
22	Śreniawitów (Sq)	18.70 ± 1.87	16.56 ± 1.66	15.88 ± 1.59
23	Targowa (S)	18.77 ± 1.88	17.78 ± 1.78	17.13 ± 1.71
24	Witosa-Okulickiego-Krakowska (C)	18.86 ± 1.89	16.81 ± 1.68	15.97 ± 1.60
25	University Rejtana (S)	18.74 ± 1.87	17.82 ± 1.78	16.24 ± 1.62
–	Bortle class	VI	IX	IX

The measurements were carried out using the Sky Quality Meter (SQM-L) photometer. In addition, the percentage increase or decrease in sky brightness in 2021 compared to 2015 was calculated. An arrow pointing up means that the surface brightness of the night sky has increased, while an arrow pointing down means that the surface brightness of the night sky has decreased. The following designations have been adopted: (R)—roundabout, (C)—crossroads, (S)—street, (A)—avenue, (Sq)—square. The measurement results for 2015 and 2018 come from the paper^5^.

**Table 3:** Sky brightness measurements in neighboring towns.

No.	Location	Brightness (mag/arcsec^2^)		
		2015	2018	2021
1	A4 Highway	20.30 ± 2.03	18.52 ± 1.85	18.11 ± 1.81
2	Biała	20.01 ± 2.00	18.84 ± 1.88	18.45 ± 1.84
3	Boguchwała	20.39 ± 2.04	18.26 ± 1.83	18.65 ± 1.87
4	Bzianka	19.68 ± 1.97	18.64 ± 1.86	18.33 ± 1.83
5	Kielnarowa	20.85 ± 2.09	18.90 ± 1.89	18.73 ± 1.87
6	Krasne	20.05 ± 2.01	17.57 ± 1.76	17.44 ± 1.74
7	Malawa	20.49 ± 2.05	19.54 ± 1.95	19.29 ± 1.93
8	Matysówka	20.96 ± 2.10	20.22 ± 2.02	20.01 ± 2.00
9	Miłocin	19.70 ± 1.97	18.81 ± 1.88	18.45 ± 1.85
10	Przybyszówka	19.60 ± 1.96	17.67 ± 1.77	17.90 ± 1.79
11	Rogoźnica	20.40 ± 2.04	18.93 ± 1.89	18.55 ± 1.86
12	Słocina	19.51 ± 1.95	19.92 ± 1.99	19.75 ± 1.98
13	Trzebownisko	19.95 ± 2.00	18.50 ± 1.85	18.22 ± 1.82
14	Tyczyn	20.52 ± 2.05	18.93 ± 1.89	18.88 ± 1.89
15	Zaczernie	20.18 ± 2.02	18.43 ± 1.84	18.37 ± 1.84
–	Bortle class	From IV to V	From V to VIII	From V to VIII

**Figure 6:**
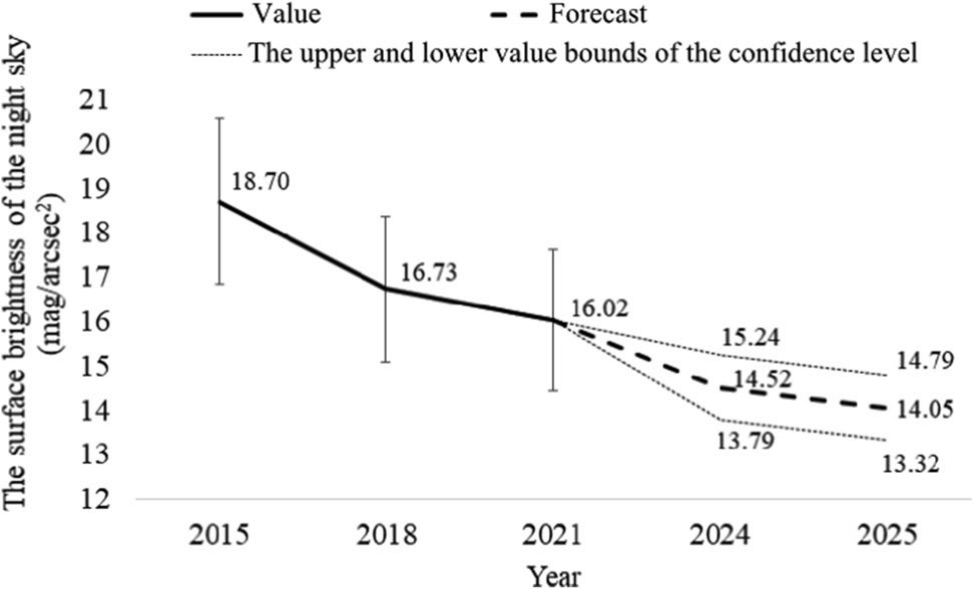
Forecast of the development of surface brightness of the night sky for the city of Rzeszów (for the market).

**Figure 7:**
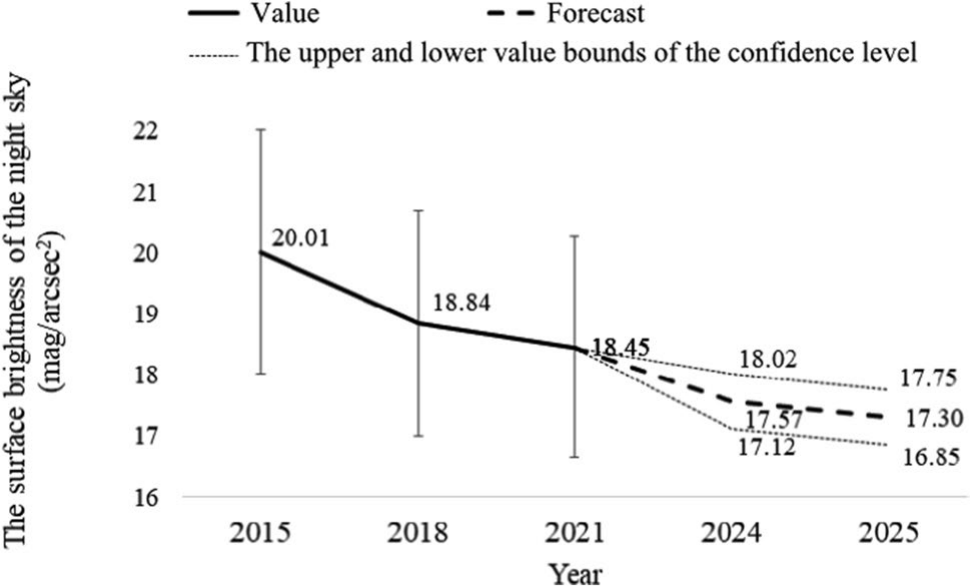
Forecast of the development of surface brightness of the night sky for the neighboring towns (e.g. Biała).

Based on the measurement results and the Excel spreadsheet, a forecast of the development of the surface brightness of the new sky until 2025 was prepared. The forecast assumes that the confidence level α = 95%, which means that 95% of the results fall between the upper and lower limits of the forecast. The results of this analysis for the city of Rzeszów (for the market) are presented in Fig. 6 and for other locations in Table 4. Whereas, in the case of neighboring towns, e.g. Biała, in Fig. 7 and for other locations in Table 5.

**Table 4:** Forecast value of the night sky's surface brightness and lower and upper limits of the confidence level for 2025 for the city of Rzeszów.

No.	Location	Brightness (mag/arcsec^2^)		
		Forecast	Lower limit	Upper limit
1	Armii Krajowej-Krzyżanowski (R)	14.71	14.43	14.99
2	Bat. Chłopskich-Langiewicza (C)	13.05	12.57	13.52
3	Chopin (S)	13.14	12.54	13.73
4	Ciepliński (A)	14.13	13.37	14.90
5	Dąbrowskiego-Wincentego Pola (C)	12.46	12.06	12.86
6	Dębicka (S)	14.57	14.27	14.88
7	Grunwaldzka-Piłsudski (C)	15.04	14.86	15.22
8	John Paul II (R)	13.82	13.02	14.61
9	Kopisto-Niepodległości-Rejtana (C)	13.79	12.70	14.87
10	Kopisto-Podwisłocze (C)	13.94	13.35	14.53
11	Krakowska (S)	14.25	13.64	14.86
12	Kuroń (R)	15.51	15.15	15.88
13	Mazowiecki Bridge	15.83	15.37	16.29
14	Pakosław (R)	13.69	12.93	14.44
15	Piłsudski (A)	13.56	12.62	14.49
16	Pobitno (R)	14.31	13.49	15.14
17	Powstańców Warszawy (A)	14.64	14.06	15.22
18	Powstańców Warszawy-Dąbrowskiego (C)	13.60	12.63	14.58
19	Rejtana-Lwowska-Dworaka (C)	14.30	13.48	15.11
20	Rejtana-Powstańców Warszawy (C)	13.81	12.51	15.12
21	Rzeszów Market (Sq)	14.05	13.32	14.79
22	Śreniawitów (Sq)	13.79	12.94	14.64
23	Targowa (S)	15.99	15.79	16.19
24	Witosa-Okulickiego-Krakowska (C)	13.87	13.17	14.57
25	University Rejtana (S)	14.26	13.72	14.80

**Table 5:** Forecast value of the brightness of the night sky surface and the lower and upper limits of the confidence level for 2025 for neighboring towns.

No.	Location	Brightness (mag/arcsec^2^)		
		Forecast	Lower limit	Upper limit
1	A4 Highway	16.45	15.66	17.25
2	Biała	17.30	16.85	17.75
3	Boguchwała	17.13	15.67	18.59
4	Bzianka	17.33	16.90	17.75
5	Kielnarowa	17.06	16.03	18.10
6	Krasne	15.37	14.00	16.73
7	Malawa	18.39	17.98	18.80
8	Matysówka	19.30	18.99	19.61
9	Miłocin	17.54	17.23	17.85
10	Przybyszówka	16.46	15.20	17.71
11	Rogoźnica	17.16	16.53	17.79
12	Słocina	19.99	19.66	20.33
13	Trzebownisko	16.90	16.22	17.58
14	Tyczyn	17.57	16.67	18.46
15	Zaczernie	16.92	15.94	17.90

## Discussion

The increase in sky pollution with artificial light entails many negative factors relating to the entire environment. In particular, this applies to astronomy in the context of planning and conducting direct astronomical observations. The consequence of the increase in the surface brightness of the night sky is the increase in the range of the light island, i.e. the scattering of artificial light in the atmosphere and its impact on the quality of the night sky, even in neighboring towns. In this case, there is an accumulation of sky pollution caused by two factors. The first is the pollution of the sky caused by natural light, e.g. from the moon. The second factor is the pollution of the sky caused by artificial light. All this means that the range of diffused light is still recorded at a distance of up to 25 km from Rzeszów.

Analyzing the obtained results, it is easy to see how much the condition of the night sky in the city center and surrounding villages has deteriorated. In the case of the city of Rzeszów, the increase in sky brightness changed the Bortle scale from class VI in 2015 to class IX in 2021. Measurements show that over six years, the brightness of the night sky in the city of Rzeszów increased by an average of 14.37%. The neighboring towns also recorded an increase in the brightness of the night sky from class IV in 2015 to class VIII in 2021. In this case, the average value of the brightness of the night sky increased by 7.74%. The percentages given for this trend apply to the same types of lighting.

Analyzing the individual percentage results (see Figs. 4, 5), we can see that in the case of the city of Rzeszów, at each measurement point, an increase in the surface brightness of the night sky was obtained in subsequent research periods. On the other hand, in the case of neighboring towns, an upward trend was observed in the vast majority of measurement points. A certain exception to this rule was three measurement points: Boguchwała (No. 3 in Table 3) and Przybyszówka (No. 10 in Table 3), for which a decrease in the surface brightness of the night sky was recorded in 2021. However, in the case of one neighboring town—Słocina (No. 12 in Table 3), a different trend was recorded, i.e. a decrease in the surface brightness of the night sky in 2018 and 2021 compared to 2015. The direct cause of these decreases is believed to be the partial replacement of lighting, both street lamps and the lighting of some buildings. Analyzing the obtained results of measurements of the surface brightness of the night sky, it can be noticed that in the vast majority of cases, the results were consistent with the global trend, which is the increase in the surface brightness of the night sky^26^. Unfortunately, this tendency causes visual observations of celestial bodies to be severely limited by even a few per cent each year. Let us note that a recently published analysis of this data found that the average night sky got brighter by 9.6% per year from 2011 to 2022, which is equivalent to doubling the sky brightness every 8 years^27^.

Based on the presented results of forecasts for the development of artificial light pollution, it can be concluded that we will most likely still have to deal with an increase in the brightness of the night sky. In the case of the city of Rzeszów in 2025 (for the market), the value of the forecast brightness of the night sky will most likely be approximately 14.05 mag/arcsec^2^. However, in the case of neighboring towns (e.g. Biała), the brightness of the sky will be 17.30 mag/arcsec^2^. Of course, at this point, you should also remember the confidence level of the forecast, i.e. the upper and lower limits of the forecast. For the city of Rzeszów (for the market) for 2025, the lower forecast value is 13.32 mag/arcsec^2^, and the upper forecast value is 14.79 mag/arcsec^2^ (see Fig. 6). However, in the case of neighboring areas (e.g. Biała), the lower forecast value is 16.85 mag/arcsec^2^, and the upper one is 17.75 mag/arcsec^2^ (see Fig. 7). Using the measurement results in all locations, the forecast value of the surface brightness of the night sky was determined, along with the lower and upper limits of the confidence level for 2025. This summary is presented in Tables 4, 5. Due to the forecasted further increase in the brightness of the night sky, it should be expected that observers in neighboring towns will also experience changes in the quality of the sky, which will undoubtedly make astronomical observations more difficult.

Taking into account the above measurement results, it should be noted that the observations of faint astronomical objects require the observer to use at least basic observation devices. Unfortunately, along with the economic development of a given region, the quality of the sky deteriorates, which directly translates into a decrease in the visibility (with the naked eye) of some celestial bodies, e.g. comets. However, in the case of some comets, there is an extremely spectacular phenomenon which is a sudden increase in their brightness, which in the literature is referred to as a cometary outburst^28–30^. As a result of the outburst, the cometary brightness increases rapidly, usually by several magnitudes over several hours^31,32^. For a given comet to be observed with the naked eye, e.g. in the city center, the change in its brightness must be significant. Such an example was the famous comet 17P/Holmes, which brightened from 16.5 to 2.6 magnitude on the night of October 23/24, 2007 and was visible to the naked eye from the center of the city. Let us add that comet 17P/Holmes briefly became the largest object in the solar system as a result of the outburst, as its coma expanded to a diameter greater than that of the Sun although its mass remained small^16^. The aforementioned case of comet 17P/Holmes was the most spectacular outburst that could be observed in the night sky so far.

When planning astronomical observations, in particular in the case of comets, areas should be selected that are relatively free of light pollution, e.g. the Bieszczady Dark Sky Park. Let us emphasize that this is one of the last regions in Europe where conditions are favorable for conducting direct astronomical observations.

## Data Availability

The sole author, all data generated or analysed during this study are included in this published paper.
